# Heterozygotic *Brca1* mutation initiates mouse genome instability at embryonic stage

**DOI:** 10.1038/s41389-022-00417-3

**Published:** 2022-07-22

**Authors:** Xiaobing Wu, Maoni Guo, Jian Cui, Haoyang Cai, San Ming Wang

**Affiliations:** 1 MOE Frontiers Science Center for Precision Oncology, Institute of Translational Medicine, Faculty of Health Sciences, University of Macau, Macau, China; 2 Eppley Cancer Institute, University of Nebraska Medical Center, Omaha, NE USA; 3 Center of Growth, Metabolism, and Aging, Key Laboratory of Bio-Resources and Eco-Environment, College of Life Sciences, Sichuan University, Chengdu, China

**Keywords:** Genomic instability, Breast cancer

## Abstract

*BRCA1* mutation is the genetic predisposition in causing genome instability towards cancer. *BRCA1* mutation is predominantly germline inherited at the fertilization. However, when the inherited mutation initiates genome instability in the mutation carriers remains largely elusive. We used a heterozygotic *Brca1*-knockout mouse as a model to investigate the issue. Through whole-genome sequencing and bioinformatics analysis, we monitored genome status across the developmental stages from embryo to adulthood in the mouse model. We observed that genome instability as reflected by structural variation, indel and copy number variation already appeared at 10.5-day embryo and progressively towards adulthood. We also observed that the genome instability was not linearly accumulated but dynamically changed along the developmental process, affecting many oncogenic genes and pathways including DNA damage repair, estrogen signaling, and oncogenesis. We further observed that many genome abnormalities in the cancer caused by *Brca1* mutation were originated at embryonic stage, and *Trp53* (*TP53*) mutation was not essential for the *Brca1* mutation-caused genome instability in the non-cancer cells. Our study revealed that heterozygotic *Brca1* mutation alone can cause genome instability at embryonic stage, highlighting that prevention of *BRCA1* mutation-related cancer in humans may need to start earlier than currently considered.

## Introduction

*BRCA1* is essential for maintaining genome stability by repairing double-strand DNA breaks through homologous recombination (HR) [1–3]. However, human *BRCA1* is also vulnerable to germline mutation due largely to the positive selection specifically imposed in human *BRCA1* [4, 5]. The mutated BRCA1 impairs its function of repairing double-strand DNA breaks, leading to genome instability, cellular transformation, and eventually cancer effecting mostly breast and ovarian [6, 7]. Nearly all human *BRCA1* germline mutation carriers are heterozygotes as *BRCA1* homozygotic mutation is embryonic lethal [8].

Although the germline nature of *BRCA1* mutation determines that the mutation is inherited at fertilization, it can take decades for the mutated BRCA1 to transform normal cells into cancer cells [9]. Taking the advantage of longer cancer-free time, early cancer prevention for the mutation carriers can be achieved if the *BRCA1* mutation-caused transformation process can be blocked before cancer development. However, most studies on *BRCA1* mutation-caused genome instability focused on the already transformed cancer cells [10–15]. As such, the current knowledge on *BRCA1* mutation-caused genome instability reflects basically the consequence of *BRCA1* mutation-caused genome instability. How *BRCA1* mutation-caused genome instability develops from the non-transformed cells to the transformed cancer cells remains largely elusive. Lack of the knowledge of early genome instability hampers the proper time to take preventive actions to minimize cancer risk for the mutation carriers.

We hypothesized that *BRCA1* mutations can cause genome instability far ahead of cellular transformation. We reasoned that by dynamically monitoring genome status in *BRCA1* mutation carriers during the developmental process before cancer development, we would be able to test our hypothesis. We considered that Brca1-mutated mouse model will be ideal for the study as mouse model has been widely used to study the relationship between *Brca1* mutation and cancer [16]. In current study, we used an established heterozygotic *Brca1* exon11-knockout mouse as the model [17]. Through whole-genome sequencing and bioinformatic data analysis, we traced the status of genome stability from embryo to adulthood (Fig. S1). Data from our study revealed that heterozygotic mutated *Brca1* initiates genome instability at the early embryonic stage.

## Results

### Experimental design

We collected genomic DNA from *Brca1*+/− mice at different developmental time points from embryo to adulthood. We performed whole-genome sequencing for each DNA sample, analyzed genomic sequences to search for the evidence of genome instability represented by SV, Indel and CNV, and compared the data between different time points. We also generated *Brca1*+/− *Trp53*+/− mice, collected and sequenced the DNA at the same time points, and compared the variation data between *Brca1*+/− mice and *Brca1*+/− *Trp53*+/− mice (Fig. S1).

### Genome instability appeared at embryonic stage and dynamically changed

**Fig. 1:**
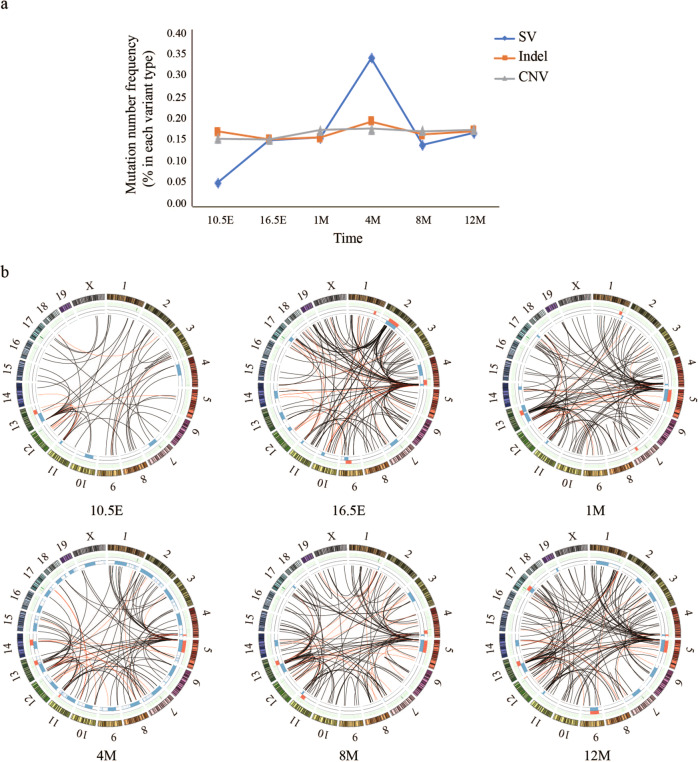
Monitoring genome instability across developmental stages. *Brca1*+/− mice in 10.5E, 16.5E, 1M, 4M, 8M and 12M (*n* = 2 in each group) were tested. **a** SVs, Indels, and CNVs at different time points. Data from 2 mice in each group were combined and divided by 2 to obtain the average value. *X*-axis: the time points. Y-axis: the mutation frequency normalized by the median. **b** Representative Circos plots showing the variation at different time points. Outer: indels; middle: CNVs (red: gain; blue: loss); inner: SVs (red: gene affected; black: no gene affected).

**Fig. 2:**
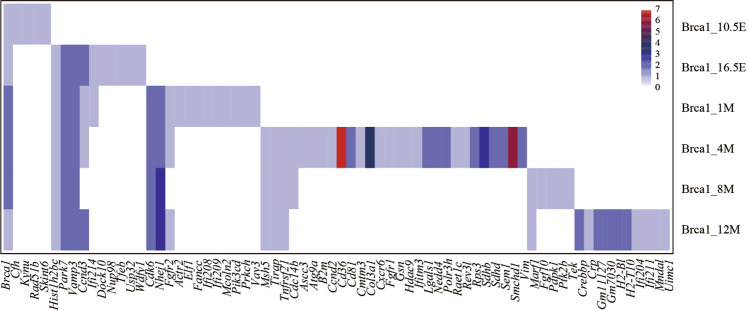
Dynamic changes of functionally important genes disrupted by SVs from embryonic towards adulthood stages. Heatmap showed the functionally important genes affected by SVs in *Brca1*+/− mice. Mutation frequency in the genes at each time point was represented in color gradient ranging from blue to red. It shows that certain disruptions generated at early developmental stage were constantly present across the entire developmental stage, whereas others were only present at given time point(s). *Brca1* mutation (at the left) was present at each time point.

To monitor genome stability across the developmental stages, we performed whole-genome sequencing in the DNA samples in *Brca1*^+/−^ mice from 10.5 and 16.5 embryonic days to adulthood at 1st, 4th, 8th and 12th months after birth. We performed bioinformatics data analysis to identify genetic changes in the sequence data in each DNA sample. We observed that SVs, CNVs, and indels were already present at the 10.5 embryonic day in the *Brca1*^+/−^ mice, with multiple clusters present in different chromosomes (Fig. 1a, Table S2, S3). The variations changed dynamically, some were intensified, others were diminished and/or intensified again along developmental process. For example, the SV cluster chr.9: 70628040-79756364 appeared at 16.5 embryonic days, intensified at 4th months then nearly disappeared afterwards; the SV cluster chr2: 38128829–41439173 appeared at 10.5 embryonic days, intensified at 16.5 embryonic days, then disappeared at the 1st and 4th month but appeared again at the 12th month (Fig. 1b). At the gene level, the mutations affecting *Hist1h2bc, P7, Vamp3, Cdk6, Nj1, Msh5he, Tirap, Tfrsf21, Marf1* were only present at specific developmental time points, whereas the mutations affecting *Ccnd3, Fgfr2* appeared at early time, disappeared at the 8th month, and re-appeared at the latter time (Fig. 2).

**Fig. 3:**
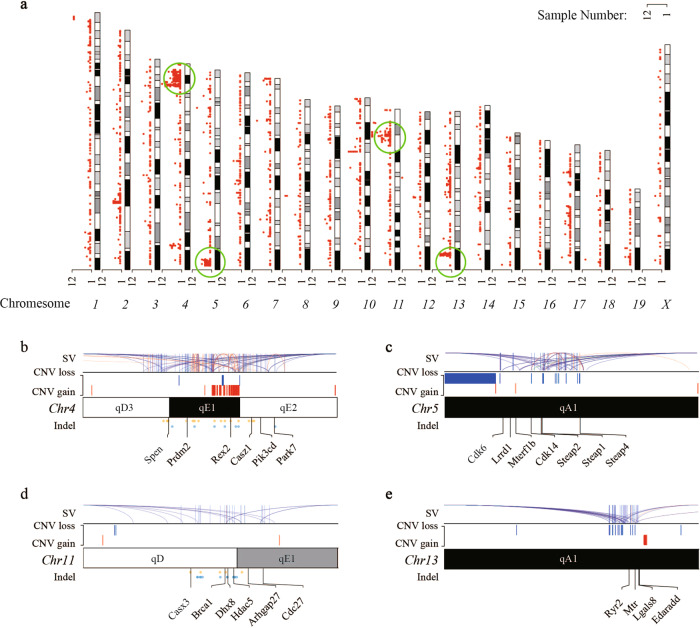
Variant distribution across different chromosomes. **a** Distribution of SV breakpoints in *Brca1*+/− mice. Red dot represents the frequency of breakpoint occurrence in the corresponding site. Green circle marks the clustered region. Variant distribution of *Brca1*+/− mice in the four major SV clusters detailed in (**b**–**e**). (**b**) chr4: 139320925–151922486; (**c**) chr5 3152512-8342821; (**d**) chr11: 96075557-107346908; (**e**) chr13: 111440505-3139770. Each cluster shows SVs [purple: break end (BND); orange: inversion; red: duplication; blue: deletion)], CNVs (blue: loss; red: gain) and Indels (yellow: insertion; blue: deletion). The curves in SVs refer to their interaction with other genomic regions. Chromosomal bands are indicated in each cluster. Representative genes affected are listed at the bottom of each cluster.

We compared the mutation distribution and identified multiple mutation hot-spots of SV, CNVs and Indels across the genomes, as represented by the four clusters of chr4: 139320925-151922486, chr5: 3152512-8342821, chr11: 9607557–107346908, and chr13: 11440505-3139770 (Fig. 1b, Fig. 3). This pattern was not present in wild-type control *Brca1*^+/+^ mice (Fig. S2, Table S4), highlighting that the changes in the *Brca1*-knockout mice were unlikely derived from background variation.

### Genome instability targeted repetitive sequences and fragile sites

**Fig. 4:**
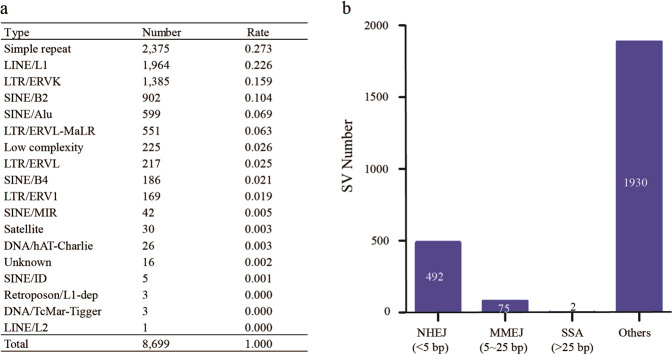
Repetitive sequences and break repair by error-prone non-homologous repair pathways. **a** Repetitive sequence classification identified at SV breakpoints sites in *Brca1*+/− mice. **b** Number of SV breakpoints repaired by error-prone non-homologous pathways of NHEJ, MMEJ, and SSA. It shows that NHEJ contributed the majority of the error-prone repairs.

We analyzed the sequences at the SV breakpoint sites in *Brca1*+*/−* mice to determine the type of sequences susceptible to the damage. The results showed that 54% of SV break sites were located at repetitive sequences of simple repeats, LINE/L1, and LTR/ERVK. The rate was much higher than the 45% of the repetitive sequences in the mouse genome (Fig. 4a) [18]. Multiple chromosomal fragile sites including Astn2, Il1rapl1, Rev3l, Thsd7a and Wwox were also present at the breakpoint sites (Table S5) [19]. The results indicated that repetitive sequences and fragile sites were vulnerably attacked by the heterozygotic *Brca1* mutation-caused genome instability.

### Genome instability promoted the use of error-prone no-homologous repair pathways

Brca1 mutation damages the error-free homologous recombination (HR) pathway but promotes the use of error-prone non-homologous end joining (NHEJ) pathways to repair double-strand DNA breaks [20]. We analyzed micro-homogenous bases at both ends of SV breakpoint sites to assess the effects of heterozygotic Brca1 mutation on non-homologous repair pathways. Based on the presence of micro-homologous bases (NHEJ 1–5 bp, MMEJ (microhomology-mediated end joining) 6–25 bp, and SSA (single-strand annealing) > 25 bp) [21–23], we identified 569 repaired double-strand break events by the non-homologous repair pathways, including 492 in NHEJ, 75 in MMEJ, and 2 in SSA (Fig. 4b, Table S6). The enrichment of NHEJ, MMEJ, and SSA-repaired damage implied that the defects in error-free homologous recombination function caused by *Brca1* mutation indeed promoted the use of error-prone non-homologous DNA repair pathways to repair the damaged double-stranded DNA, which further enhanced *Brca1* mutation-caused genome instability.

### Genome instability affected functionally important genes and pathways

**Fig. 5:**
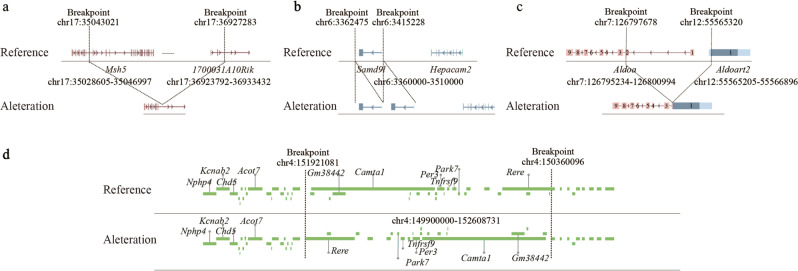
Examples of SV-disrupted genes. **a** A deletion between *Msh5* and *1700031A10Rik* at the 4th month formed *Msh5-1700031A10Rik* out-of-frame fusion. **b** A duplication in *Samd9* occurred at 16.5 embryonic days. **c** A t(7:12) translocation at the 4th month formed an out-of-frame *Aldoa-Aldoart2* fusion. **d** Normal *Rere* structure VS the inversion at 16.5 embryonic days disrupted *Rere* structure.

Overall, the genome instability by deletion, duplication, translocation, inversion caused by SVs, indels, and CNVs at different developmental stages affected over 2,300 genes in the *Brca1*^+/−^ mice genomes. Many of these affected genes are functionally important involving in oncogenesis, tumor suppression, DNA damage repair, and immune function (Table S7a, S7b). For example, Msh5 is involved in DNA mismatch repair and meiotic recombination [24]. A deletion between *Msh5* and *1700031A10Rik* at the 4th month formed *Msh5*-1700031A10Rik out-of-frame fusion; Samd9 is a tumor suppressor involving in cell proliferation and innate immune response to viral infection [25]. A duplication in *Samd9* occurred at 16.5 embryonic days; Aldoa plays a role in glycolysis and gluconeogenesis [26]. A t(7:12) translocation at the 4th month formed an out-of-frame *Aldoa*-*Aldoart2* fusion; Rere is involved in apoptosis. An inversion at 16.5 embryonic day disrupted *Rere* structure (Fig. 5); Rad51b is critical for double-stranded DNA break repair in the homologous recombination pathway [27]. A t(12:14) translocation at the 4th month formed *Rad51b*-*Fbxo34* fusion; Ccnd3 regulates G1/S transition and is frequently dysregulated in many cancer types [28]. A t(4:17) translocation at 16.5 embryonic day disrupted *Ccnd3*; Fgfr2 has tyrosine kinase activity and is frequently mutated in cancer [29]. A t(7:11) translocation at the 4th month disrupted *Fgfr2*; Hdac9 regulates histone deacetylation [30]. A t(7:12) translocation at the 4th month formed *Sptbn4*-*Hdac9* fusion; Elf1 is a transcriptional factor [31]. A frameshift insertion at the 4th month disrupted *Elf1*; Pik3cd phosphorylates inositol lipids in immune response [32]. A t(4:8) translocation at the 1st month formed *Pik3cd*-*Wwox* fusion; B2m is an MHC class I protein playing key roles in antigen presentation [33]. Inversion of the *B2m* at the 4th month disrupted *B2m*. Many mutations were located in non-coding regions. For example, there were three inversions formed in the intron 5 of *Pax7*, a gene involved in developmental regulation, between 16.5 embryonic day and 12th month (Table S7c). The functional significance of these mutations remains to be determined.

KEGG pathway analysis revealed that the affected genes were enriched in multiple oncogenesis-related pathways, including estrogen signaling (Adcy2, Adcy3, Adcy6, Akt3, Atf2, Calm1, Ctsd, Gnai2, Gnas, Hsp90ab1, Itpr1, Kcnj5, Kcnj6, Krt10, Krt13, Krt20, Mmp2, Ncoa2, Pik3ca, Pik3cd, Plcb4, Sos1), cell cycle regulation (Anapc13, Ccnd2, Ccnd3, Cdc14b, Cdk6, Cdc27, Crebbp, Mcm7, Prkdc, Smad4, Smc1b, Stag1, Tfdp2, Ywhae, Zbtb17), cancer development (Akt3, Cdk6, Dvl2, Fgfr1, Fgf10, Mtor, Pik3ca, Pik3cd, Sos1), DNA damage repair (Brca1, Rad51, Rad51b, Rad51c, Sem1, Uimc1), Fanconi anemia (Rad51, Rad51c, Rev1, Rev3l), and base excision repair (Pole2, Pole3, Pole4) (Fig. S3, Table S7d). The abundant genes and pathways affected by the genome instability provided an environment in promoting cellular transformation towards cancer.

### Certain genome instability in cancer cells originated at embryonic stage

Taking advantage of the genome instability data available from the cancer developed in the same *Brca1*+*/−* mice [Fig. 1 in ref. 34], we compared the data between the non-cancer observed in our study and the cancer in *Brca1*-knockout mice. The results showed that the four major SV clusters in chromosome 4, 5, 11, and 13 observed in our study largely overlapped with those in the cancer cells, as exampled by the 11qD-qE cluster shared between the 10.5-day embryo and the cancer cells (Fig. 3). The overlaps indicated that these abnormalities in the cancer cells were likely originated earlier before the transformation of non-cancer cells into cancer cells.

### The accumulated and de novo mutations

While the results above showed that the mutations were detected at the early embryonic stage, possibility may exist that the mutations detected could also include these accumulated from previous generations in the mutant strain considering that the mutant was generated more than two decades ago and propagated for many generations [17]. To test this possibility, we generated the *Brca1* mutant and *Brca1* normal mice by crossing the male and female mice of the same batch. We then sequenced the genomes at 10.5 and 16.5 embryonic days, and 1st month after birth. By using the sequences from kidney DNA, which is considered more stable than other tissue types, of the *Brca1* mutant as the filter, we separated the accumulated mutations from the *de novo* mutation. We observed that around 2/3 of the mutations was the accumulated mutations (Table S8) and 1/3 were the de novo SVs, Indels, and CNVs, with SVs in particular, with similar patterns observed above as reflected by hotspot mutation formation, dynamic mutation change along developmental stages, breakpoints located at repetitive sequences (Table S9), affected genes including oncogenes, tumor suppressors, DNA damage repair genes, and immune function genes (Table S10), and mutated genes in non-homologous repair pathways (Table S11). The presence of de novo mutations after removing the accumulated mutations in the mutant mice confirmed that genome instability was indeed present at the embryonic stage in the heterozygotic *Brca1* mutant genome.

### Trp53 mutation played limited roles in Brca1 mutation-caused genome instability

Previous studies in the cancer developed in *Brca1* mutant mice showed that Trp53 (TP53) mutation was required for the mutated *Brca1* to cause genome instability [35, 36]. We introduced the *Trp53*+/− mutation to *Brca1*+/− to generate the *Brca1*+/− *Trp53*+/− mice. Using whole-genome sequencing, we collected the mutation data from *Brca1*+/− *Trp53*+/−, and compared the mutation data between *Brca1*+/− and *Brca1*+/− *Trp53*+/− mice. The results showed no significant differences for SVs and CNVs between the two groups but certain differences in indel (Table S12), indicating that Trp53 mutation was not essential for heterozygotic *Brca1* mutation-caused genome instability in non-cancer cells.

## Discussion

Because homozygotic *BRCA1/Brca1* germline mutation is embryonic lethal, nearly all *BRCA1/Brca1* mutation-related patients are heterozygotic. Therefore, the genome instability caused by the *BRCA1/Brca1* mutation basically refers to the heterozygotic *BRCA1/Brca1* mutation-caused genome instability. In this study, we analyzed the genome status in the heterozygotic *Brca1*-mutated mice across the developmental process from embryonic towards adulthood.

Previous studies showed that the genome instability in BRCA1/Brca1-mutated cancer included chromosome rearrangement-affected tumor suppressor genes such as TP53 causing cancer progression. However, it remains largely unclear for the genome-wide patterns of genome instability in the BRCA1/Brca1 mutation carriers, particularly the dynamic features along the developmental process before cellular transformation. Our study showed that genome instability as reflected by SVs, Indels and CNVs were already present at the 10.5 embryonic day.

It is interesting to note that the genome instability observed at the early embryonic stage did not progress linearly but dynamically along the developmental process. While the mechanism remains unclear, it is possibly related with the dosage relationship between the intact copy expressing the wild-type Brca1 to repair the damaged DNA and the mutated copy expressing the mutated Brca1 unable to repair the damaged DNA. It is known that the expression of the mutated BRCA1 is low in breast and ovarian cancer [37]. The periodic expression of Brca1 alone developmental stages further complicated the dosage relationship between the wild-type and the mutated Brca1 copies [38]. As coordination between HR and NHEJ is essential to repair double-strand DNA damage, the high events of NHEJ, MMEJ, and SSA imply that the damaged HR by *Brca1* mutation increased the use of error-prone NHEJ, MMEJ, and SSA pathways to repair the double-strand DNA damage and further enhanced the genome instability. These factors may jointly contribute to the early genome instability observed in the *Brca1* mutant.

Besides genomic instability spread across the genome, we also observed the presence of mutation hot-spots in the Brca1+/− mice affecting many genes. This indicated that the heterozygotic *Brca1* mutation-caused genome instability was not randomly distributed but under certain selection. It is known that repetitive sequences and fragile sites play important roles in genetic instability [39]. Data from our study *revealed* confirmed that repetitive sequences and fragile sites were indeed targeted by the heterozygotic *Brca1* mutation-caused genome instability.

Numerous studies have revealed that *Brca1*-mutated tumors display extensive genetic alterations causing abnormal gene expression, abnormal estrogen signaling, and LOH [40–42]. Consistent with these observations, our study observed that bulk of genes with various important function were affected by the heterozygotic *Brca1* mutation. Many of these genes were closely related with oncogenesis, immunity and estrogen metabolism. It is interesting to note that *TP53* mutation considered as essential in *BRCA1*-mutated breast cancer cells plays limited roles in the early genome instability caused by *Brca1* mutation. This can be expected as *TP53* mutation is mostly somatic, occurring in later stage of cellular transformation.

In summary, our study made the following observations:

1. Genome instability can be initiated in *Brca1*+/− mice at the early embryonic stage towards the adulthood.
2. The genome instability can generate multiple hotspot mutation clusters in the genome.
3. Repetitive sequences and fragile sites can be vulnerably attached by the genome instability.
4. The genome instability can promote the use of error-prone non-homologous repair pathways to repair double-strand DNA damage, leading to enhanced genome instability.
5. The genome instability may not progress linearly but fractally across developmental stages.
6. The genome instability can disrupt many functionally important genes and pathways.
7. Many genome instability events in *Brca1* mutation-caused cancer cells can be originated from the early genome instability initiated in non-cancer cells.
8. Unlike the genome instability in cancer cells, TP53 mutation may not be essential for the early genome instability induced by the heterozygotic *Brca1* mutation.

Based on the observations from our study, we propose a model to explain how heterozygotic *Brca1* mutation leads to early genome instability: Heterozygotic *Brca1* mutation causes Brca1 dosage change by decreased presence of functional Brca1. This change affects double-strand DNA damage repair function soon after fertilization, causing genome instability at early embryonic stage and progressively towards the adulthood. The damaged homologous recombination pathway promoted the usage of error-prone non-homologous recombination pathways to repair double-strand DNA damages and enhanced the genome instability. The functionally important genes and pathways disrupted by the genome instability provide an oncogenic environment for cellular transformation towards cancer.

The early oncogenic effects of *Brca1* mutation highlights that cancer prevention in human *BRCA1* mutation carriers may need to start earlier than current practice in order to effectively disrupt the oncogenic process. It remains to determine whether similar situation could also exist in other cancer predisposition genes.

## Materials and methods

### Knockout mice used in the study

In our study, we used the *Brca1* heterozygous-knockout mouse (*Brca1*+/−) generated by deletion of *Brca1* exon 11 through Cre-LoxP recombination in 129S6/SvEvTac mouse [17]. We also used *Brca1* heterozygous-knockout/*Trp53* heterozygous-knockout mouse (*Brca1*+/−, *Trp53*+/−) [17] generated by crossing *Brca1*^*+/−*^ with *Trp53* ^*+/−*^ mice, in which *Trp53* exon 5 was disrupted [35]. In the control experiment for distinguishing between the generation-accumulated and the de novo mutations, we generated the *Brca1* mutant and *Brca1* normal mice by crossing the male and female mice of the same batch. A total of 21 *Brca1*^+/−^ female mice, 11 *Brca1*^*+/−*^ *Trp53*^*+/−*^ female mice, and 8 *Brca1*^+/+^ *Trp53*^+/+^ wild-type female mice were used for whole-genome sequencing analysis in the study (Fig. S1). Mice were housed under specific pathogen-free conditions at the University of Macau Animal Facilities. The study was approved by the University of Macau Animal Care and Use Committee (UMAEC/UMARE No. 041-2017).

### Genotyping

Genotyping for each mouse was performed by PCR on the condition: PCR was performed in 12.5 µl of 2×Taq PCR MasterMix (kt201, Tiangen, Beijing, China), 1 µg DNA, 0.1 µM forward primer and reverse primer, and 23 µl ddH2O. PCR reactions were run on a 7900 HT Sequence Detection System (Thermo Fisher Scientific, Waltham, MA, USA). Cycling conditions were 94 °C for 3 min, followed by 30 cycles of 94 °C 30 s, 55 °C 30 s, 72 °C 60 s, and a final cycle at 72 °C for 5 min. Five µl of PCR products were loaded on 1% of agarose gels for electrophoresis. Mice did not fit the genotype criteria (*Brca1*+/−, or *Brca1*+/− *Trp53*+/−) were excluded.

The PCR primers used for genotyping *Brca1* (Table S1):

Primer 1- F1: 5′-CTGGGTAGTTTGTAAGCATCC-3′,

Primer 2- R1: 5′-CAATAAACTGCTGGTCTCAGGC-3′,

Primer 3- R2: 5′-CTGCGAGCAGTCTTCAGAAAG-3′

The PCR primers used for genotyping *Trp53*:

Primer 1- F1: 5′-CTGTCTTCCAGATACTCGGGATAC-3′

Primer 2- R1: 5′-CCAATGGTGCTTGGACAATGTG-3′

Primer 3- R2: 5′-ATCGCCTTCTATCGCCTTCTTGACGAGTTC-3′

### DNA collection and whole-genome sequencing

The presence of plug after mating was counted as 0.5 embryonic day (0.5E). We collected genomic DNA at embryonic stages of 10.5 and 16.5 days, and adulthood stages of 1st, 4th, 8th, and 12th months after birth, with two mice at each time point, and two littermate’s wild-type mice (*Brca1*^*+/+*^ *Trp53*^*+/+*^) in 10.5E and 4M as the wild type control. Mice were selected randomly. To collect embryonic DNA samples at the 10.5E and 16.5E, pregnant mice were sacrificed by carbon dioxide suffocation. A single embryo from 10.5E and 16.5E was used for DNA extraction. To collect DNA samples after birth, mice were anesthetized by intra-peritoneal injection of Avertin (500 mg/kg), and a single mammary gland was dissected under surgical sterility condition from the same mouse at 1st, 4th, 8th, and 12th months after birth and wound was sealed after each operation. In the control experiment for distinguishing between the generation-accumulated and the de novo mutations, we collected and sequenced the DNA at 10.5 and 16.5 embryonic days, and one month after birth. DNeasy Blood & Tissue Kit (Cat. 69504, Qiagen, MD, USA) was used for DNA extraction following the manufacturer’s instructions. Briefly, tissue was grinded, 20 µl of Proteinase K and 4 µl of RNase A were added, mixed and incubated overnight at 56 °C. Lysed tissues were vortexed for 15 s, followed by adding 200 µl of Buffer AL and 200 µl of ethanol. The mixtures were transferred to the DNeasy Mini spin column, centrifuged at 8000 rpm for 1 min. Then 500 µl of Buffer AW1 and 500 µl of Buffer AW2 were added and centrifuged for 3 min at 14,000 rpm. DNA was then eluted with 200 µl of ddH_2_0, and quantified by Nanodrop 2000 (Thermo Fisher Scientific, CA, USA). DNA samples were subjected to whole-genome sequencing at pair-end 2 × 150, 30X coverage in Illumina HiSeq 2500 sequencers (Novogen, Beijing, China).

### Variant calling

Quality control was performed for all FASTQ data by FastQC (Version 0.11.5). Low-quality reads were removed by Trimmomatics (Version 0.36). Sequence reads were aligned to Mouse Genome Reference Sequences (mm10) using BWA-MEM. Unmapped reads and duplicates were removed by Picard (version 2.18.25).

SV (structural variant) of duplication (DUP), deletion (DEL), inversion (INV), and chromosomal translocation (BND) was called using DELLY v2 with default settings [43]. Variants called from wildtype control were used to remove the SV sequences different between 129S6/SvEvTac and mm10. SVs passed the quality filter were adjusted for the analysis [44]. Briefly, these with (1) poor mapping quality (median MAPQ < 40); (2) with discordant reads in paired normal files; (3) belonging to DNA library artifacts were filtered out. Breakpoint positions and microhomology sequences were detected using the “SA tag” of the clipped reads. Breakpoints were annotated by referring to mm10 using Bedtools. Circos plot and Karyoploter package were used to show the genome-wide distribution of SVs. Matplotlib package in python was used for SV clusters.

Indels were called using HaplotypeCaller in GenomeAnalysisToolkit (GATK) 4 Best Practices pipelines [45]. After GATK VairantFiltration, the results were annotated and classified as implemented in ANNOVAR. Indel data from the wildtype control mice were used to remove the Indel sequences different between 129S6/SvEvTac and mm10. Circos plot was also used to show the genome-wide distribution of indels.

CNVs (copy number variant) were called using CNVnator v0.3.3 following the instruction. The bin size for each sample was set at 100 and the following filters were used in data processing: (1) q0 below 0.5; (2) Length of the CNVs > 1 kb; (3) e-value <0.05; (4) Deletions with normalized average read depth <0.4 and duplications with normalized average read depth >1.6 [46]. CNVs called in the wildtype control were used as the filter to remove the CNV sequences different between 129S6/SvEvTac and mm10. The results were annotated by referring to mm10 using Bedtools [47]. Circos plot was used to show the genome-wide distribution of CNVs. Each type of variation data at each time point from the two mice were combined to represent the variation at each time point.

### Sequence and functional analyses

To identify the repetitive and fragile sites at SV breakpoints, two biological replications from the same time point were combined together. Fifty-bp sequences at each side of the SV breakpoints in *Brca1*+/− mice were extracted and searched against the mouse RepeatMasker genomic dataset (http://www.repeatmasker.org/) [48]. SV-affected genes from the same time point in *Brca1*+/− mice were compared with the fragile sites in the mouse reference genome to identify the genes at the corresponding fragile sites.

To Identify the double-strand break repair by non-homologous repair pathways, all SVs in *Brca1*+/− mouse were combined together and the breakpoint sites were extracted after removing the repeated ones. Fifty-bp sequences at both sides of the SV breakpoint were used to identify microhomology features based on the base number of microhomology sequences: 1–5 bp for NHEJ (non-homologous end joining), 6–25 bp for MMEJ (microhomology-mediated end joining); and >25 bp for SSA (single-strand annealing) [21–23].

For functional annotation and analysis of KEGG pathway enrichment, ClusterProfiler package in R was used, *p* < 0.05 was considered as statistical significance. The results were further showed by ggplot 2. KEGG was also used to identify functional categories of the genes affected by the mutations [49].

### Statistical data analysis

Two biological replications from the same time point were combined together. Unpaired Student’s *t* test (two-sided) in R was used to determine significant differences in genome instability between *Brca1*+/− group and *Brca1*+/− *Trp53*+/− group.

## Supplementary information

supplementary material

Figure S1

Figure S2

Figure S3

Table S1

Table S2

Table S3

Table S4

Table S5

Table S6

Table S7

Table S8

Table S9

Table S10

Table S11

Table S12

## Data Availability

The whole-genome sequence data collected in this study were deposited at the NCBI Sequence Read Archive (SRA: PRJNA725083). The source code used in sequence analysis is available in GitHub: https://github.com/xiaobing996/BRCA1_TRP53. Additional information is provided as the supplementary dataset online.
