# Supplementary material for: Heterozygotic Brca1 mutation initiates mouse genome instability at embryonic stage
Source: Oncogenesis. 2022 Jul 22;11(1):41. doi: 10.1038/s41389-022-00417-3 (PMC9307611; doi:10.1038/s41389-022-00417-3)
Supplement: Supplementary file 1 — supplementary material [file 41389_2022_417_MOESM1_ESM.docx]

**Supplementary figure legends**

**Supplementary figure 1. Workflow of the study.** a. Heterozygotic *Brca1* knockout mouse was generated by Cre-LoxP recombination system. *Brca1*+/- *Trp53*+/- mouse was generated by crossing *Brca1*+/- mouse with *Trp53* exon 5-deleted mouse. DNA was collected at given time points (10.5 embryonic day, 16.5 embryonic day, 1^st^ month, 4^th^ month, 8^th^ month, and 12^th^ month after birth). b. Scheme of data analysis. Whole genome sequences were collected from each sample. SVs, CNVs, and Indels were identified from the sequences.

**Supplementary figure** **2. Circos plots showing SVs, CNVs, and Indels in wildtype mice.** It shows the background variation in the wildtype mice and decreased variation alone the developmental process.

**Supplementary figure 3. Top 50 pathways affected by variation through KEGG analysis.** Black: numbers of genes enriched; Blue: Significance at -log10 scale (adjusted P-value).

**Supplementary TABLES**

**Supplementary table 1. Genotype validation of *Brca1* and *Trp53* deletion in all samples**

**Supplementary table 2. SVs, Indels and CNVs in *Brca1* mutant**

**Supplementary table 3. Summary of SVs in each *Brca1* mutant**

**Supplementary table 4. SVs in wildtype mice**

**Supplementary table 5. Repetitive sequences and fragile sites at SVs breaking sites**

**Supplementary table 6. SV~~s~~ breaking sites repaired by different non-homologous repair pathways**

**Supplementary table 7. Genome instability-affected genes. It shows three Pax7 intron SVs in *Brca1* mutant, and KEGG classification of genome instability-affected genes**

**Supplementary table 8. de novo SVs, Indels and CNVs in *Brca1* mutant mice**

**Supplementary table 9. Repetitive sequences and fragile sites at SV breaking sites of the de novo mutation**

**Supplementary table 10. KEGG classification of genome instability-affected genes by de novo mutation**

**Supplementary table 11. de novo SV breaking sites repaired by different non-homologous pathways**

**Supplementary table 12. Comparison of mutations between *Brca1* mutant and *Brca1/Trp53* mutant groups**
