# Supplementary figures and images for: Heterozygotic Brca1 mutation initiates mouse genome instability at embryonic stage
Source: Oncogenesis. 2022 Jul 22;11(1):41. doi: 10.1038/s41389-022-00417-3 (PMC9307611; doi:10.1038/s41389-022-00417-3)

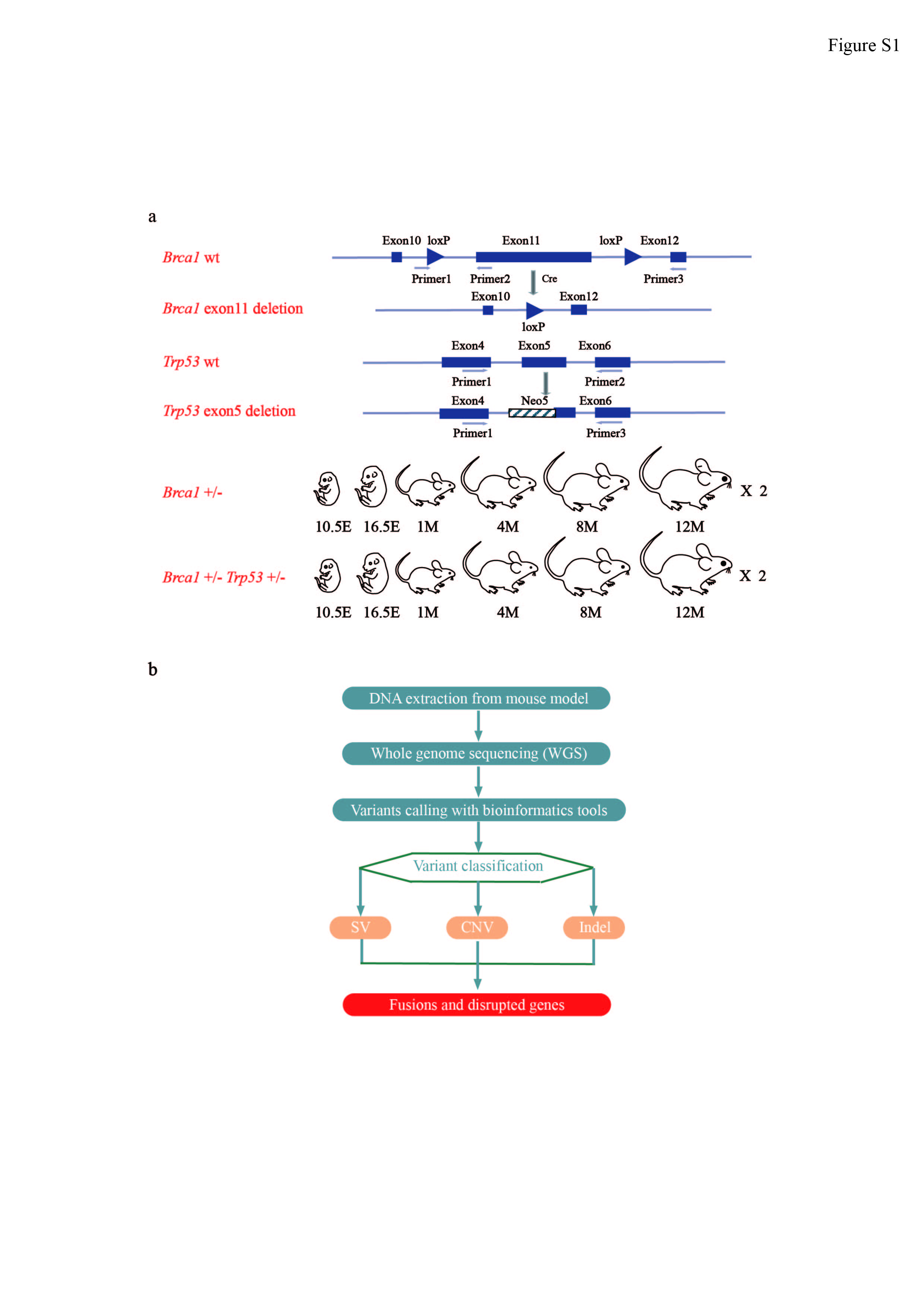

Supplement: Supplementary file 2 — Figure S1 [file 41389_2022_417_MOESM2_ESM.jpg]

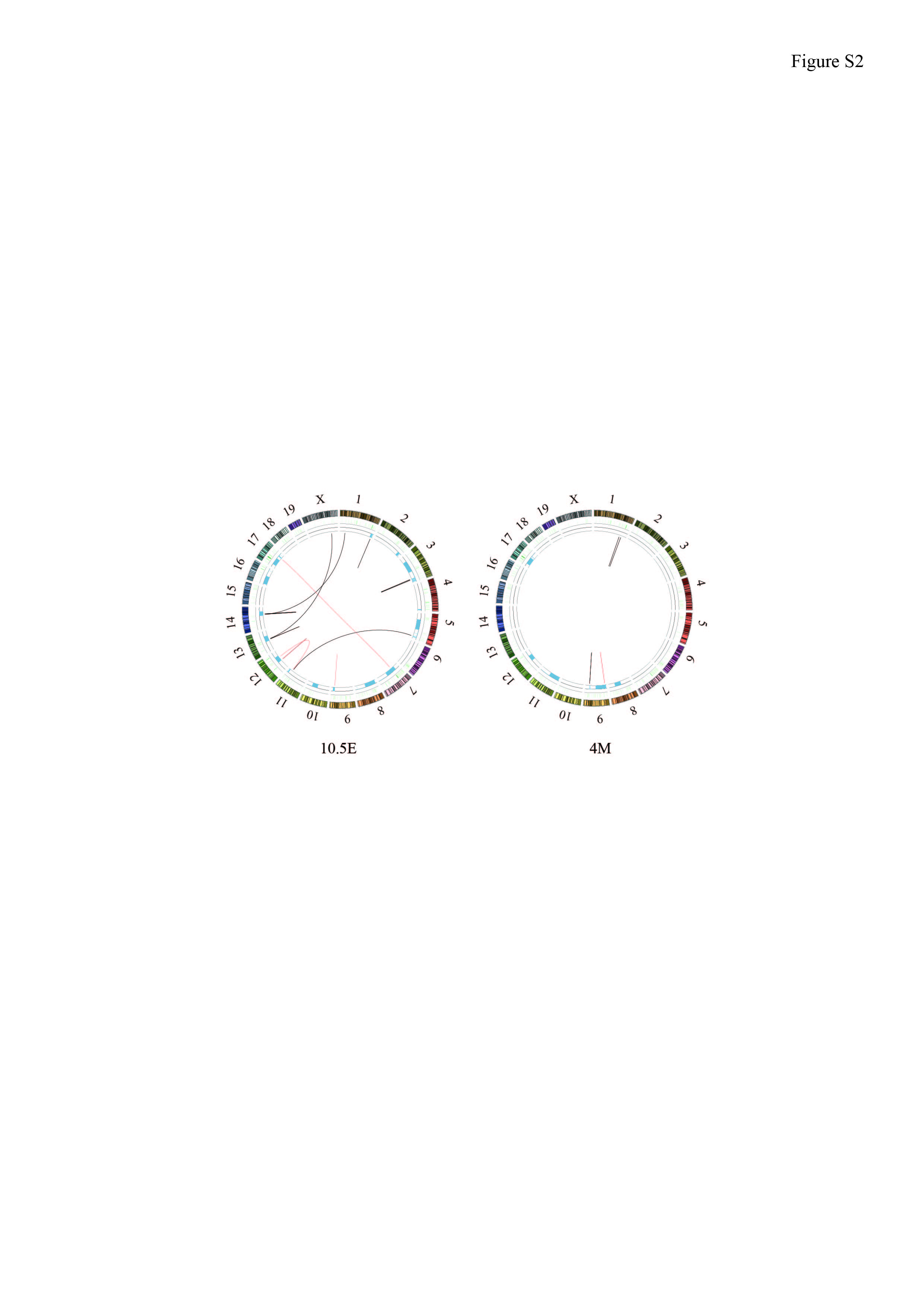

Supplement: Supplementary file 3 — Figure S2 [file 41389_2022_417_MOESM3_ESM.jpg]

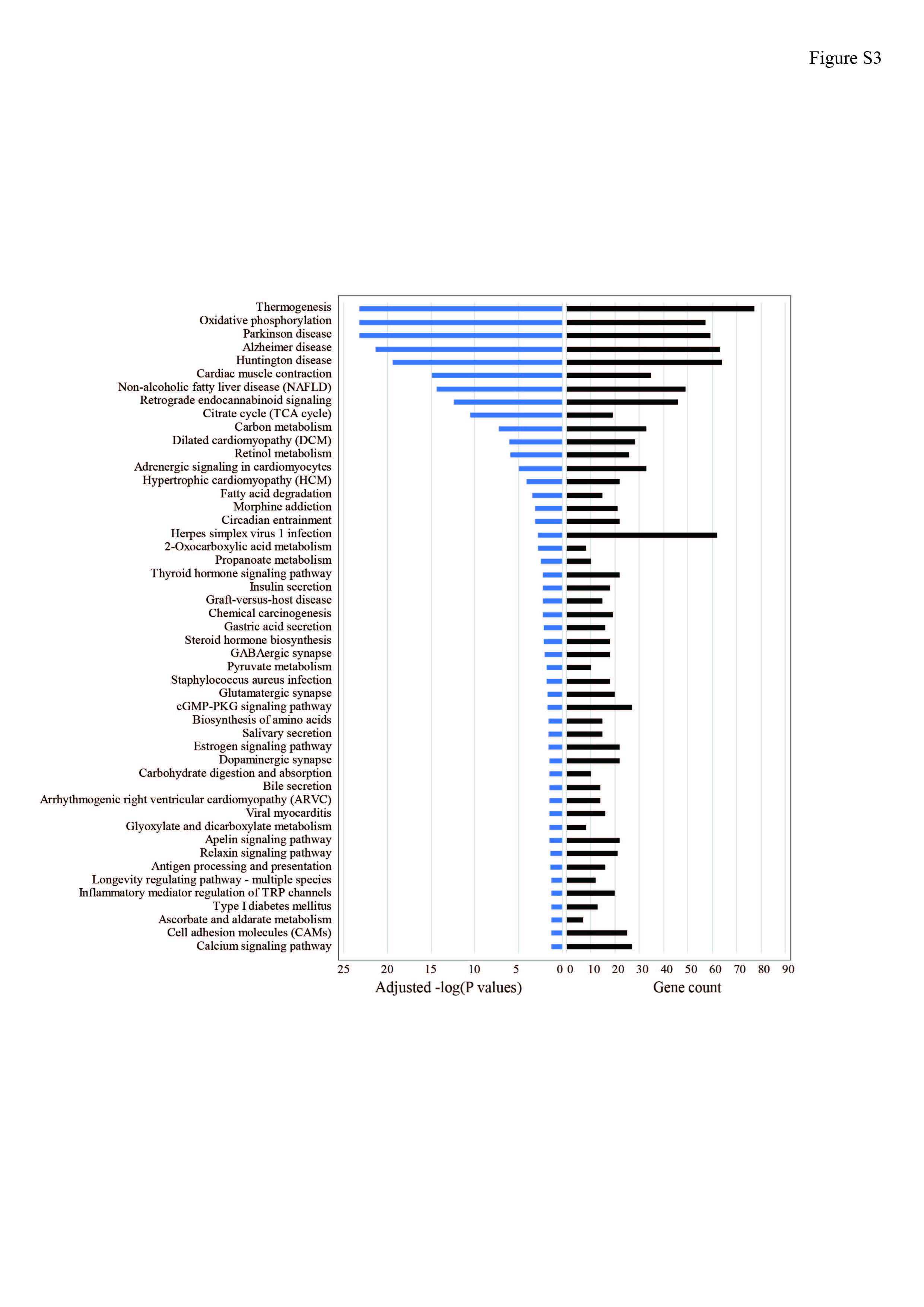

Supplement: Supplementary file 4 — Figure S3 [file 41389_2022_417_MOESM4_ESM.jpg]
